# Supplementary material for: Common Polymorphisms in the Glycoproteins of Human Cytomegalovirus and Associated Strain-Specific Immunity
Source: Viruses. 2021 Jun 9;13(6):1106. doi: 10.3390/v13061106 (PMC8227702; doi:10.3390/v13061106)
Supplement: Supplementary file 1 [file viruses-13-01106-s001.zip › viruses-1206455-supplementary.pdf]

viruses

MDPI

(a)

```
gB-1     1 MESRIWCLVVCVNLCIVCLGAAVSSSST-RGTS-ATHSHHSSHTTSAAHSRSGSVSQRVTSSQTVSHGVNETIYNTTLKYGDVVGVNTTKYPYRVCSMAQ  98
gB-2     1 MESRIWCLVVCVNLCIVCLGAAVSSSSTSHATSSTHNGSHTSRTTSA-QTRSV-YSQHVTSSEAVSHRANETIYNTTLKYGDVVGVNTTKYPYRVCSMAQ  98
gB-3     1 MESRIWCLVVCVNLCIVCLGAAVSSSSTSHATSSTHNGSHASRTTSA-QTRSV-SSQHVTSSKAVSHRANETIYNTTLKYGDVVGVNTTKYPYRVCSMAQ  98
gB-4     1 MESRIWCLVVCVNLCIVCLGAAVSSSTS-RATS-THNGNHTSHTTSA-QTRSI-SSQRVTSSEAVSHRANETIYNTTLKYGDVVGVNTTKYPYRVCSMAQ  96
gB-5     1 MESRIWCLVVCVNLCIVCLGAAVSSSSTTGSRGTSIHNHHSSHTTSAARSQSGSVSHHVTSSQTVSHDVNETIYNTTLKYGDVVGVNTTKYPYRVCSMAQ  100

gB-1   398 KLQQIFNTSYNQTYEKYGNVSVFETTGGLVVFWQGIKQKSLVELERLANRSSLNLTHNRTKRSTDGNNATHLSNMESVHNLVYAQLQFTYDTLRGYINRA  498
gB-2   398 KLQQIFNTSYNQTYEKYGNVSVFETSGGLVVFWQGIKQKSLVELERLANRSSLNITH-RTRRSTSDNNTTHLSSMESVHNLVYAQLQFTYDTLRGYINRA  497
gB-3   398 KLQQIFNASYNQTYEKYGNVSVFETTGGLVVFWQGIKQKSLLELERLANSSGVNSTR-RTKRSTGN-TTTLSPESESVRNVLYAQLQFTYDTLRSYINRA  496
gB-4   396 KLQQIFNASYNQTYEKYGNVSVFETTGGLVVFWQGIKQKSLVELERLANRSSLNLTHSRTRRSTDGTNVTHLSNMDSVHNLVYAQLQFTYDTLRGYINRA  496
gB-5   400 KLQQIFNASYNQTYEKYGNVSVFETTGGLVVFWQGIKQKSLLELERLANSSGVNATR-RSKRSTNNTTTLSL-ENDSVRSVLYAQLQFTYDTLRNYINRA  498
```

(b)

```
gN-1      1 MEWNTLVLGLLVLSVVAESSGNNSSTSTSATT-SKSSASVSTTKLTTVATTSATTTTTTTLSTTSTKLSSTTHDPNVMRRHANDDFYKAHCTSHMYELSL 99
gN-2      1 MECKTLVLGLLIISAVTGSSSSNSSTSTSST--TPSPSSVSTSKPTTSVTTSTTTTTTTTSTTSTRLSSTTHDPNVMRRHANDDFYKAHCTSHMYELSL 98
gN-3a     1 MEWKTVILGLFVLSVAAGSSGNSSSTSTSATTLKSSSSSVSTSKLTTTATTTTT-MSTTSSTTTTKPSSTTHDPNVMKRHTHDDFYKAHCTSHMYELSL 99
gN-3b     1 MEWKTRVLSFLVLSVAVGSYGNSSSTSTSASTPSPPSSSVSTVKSTTSVTTSTTPT-TTTTTLTSTKPGSTTHNPNVMKRHDHDDFYNAHCTSHMYELSL 99
gN-4a     1 MEWNTLVLGLLVLSVAASS--NNTSTVSTPSP-SSSTRTLTTVKATTTATTSTTTTRSTTSSTTSTKLSSTTHDPNVMRRHANDDFYKAHCTSHMYELSL 97
gN-4b     1 MEWNTLVLGLLVLSVAASS--NHTSTASTPSP-SSSTHTSTTVKATTTATTSTTTVTSTTSSTTSTKPGSTTHDPNVMRPHAHNDFYKAHCTSHMYELSL 97
gN-4c     1 MEWNTLVLGLLVLSVAASS--NNTSTASTPSP-SSSTRTSTTVKSTAVATTSTTTA-TSTSSTTSAKPGSTTHDPNVMRPHAHNDFYNAHCTSHMYELSL 96
```

(c)

```
gO-1a     1 MGRKEM-MVRDVPKMVFLISISFLLVSFINCKVMSKA-LYNRPWRGLVLSKIGKYKLDQLKLEILRQLET-T--ISTKY-NV-SKQPVKNLTMNMTEFPQYYILAGPIQN  103
gO-1b     1 MGRKGE-M-RGVFNLFFLMTLTFLLFSFINCRAAVRL-SVGRYWSGKVLSTIGKQRLDKFKLEILKQLEK-D--IYTKYFNM-TRQHIKNLTMNMTEFPRYYILAGPIQN  103
gO-1c     1 MGRKGD-M-RSISKLFFIISLTVLLFSIINCKVVR---PPGRYWLGTVLSTIGKQKLDKFKLEILKQLER-E--PYTKYFNM-TRQHVKNLTMNMTQFPQYYILAGPIRN  101
gO-2a     1 MGRKGE--MRGVNL-LFLIWLTFLLFFFINCKSARSQ--RA-PFRPRIWHPTVLKKLKQLKLEILRQLEP-I--PYIKYPQI-NTTRVQSLTVNMTEFPYYYILAGPIRN  100
gO-2b     1 MGKKKI-LVRGVPR-IFMV-STFLLIFLINCKGALNV-PRGRPWIGKVPSLKWKLKEQQLKIEILKQLQS-D--IYTKYPQI-TKNYTQFITTELKKFPLYYILAGPIRN  102
gO-3      1 MGRKGE-M-RGVFNLLFLISLTFLLFSLLNCKSAARV-FRLPFPYGRVLSKNGRLAEIKWKQELLKQIGASQ--DYYKFFTI-PTKQGLNAVVTMERFPDNYILAGPIQN  104
gO-4      1 MGRKGE-M-RGVFNLFFLMSLTFLLFSFINCKITVA---R-----FRVKSQKAKEEERQLKLRILQELAS-KTGDYYKFFTFPSQQKLYNITVEMKQFPPNSILAGPIRN  99
gO-5      1 MGKKEMIMVKGIPKIMLLISITFLLLSLINCNVLVNSRGTRRSWPYTVLSYRGKEILKKQKEDILKRLMS-TSSDGYRFLMYPSQQKFHAIVISMDKFPQDYILAGPIRN  109

gO-1a   267 KEQFEKKAKKTQSTTTPYFSYTTSAALNVTTNVTYSITTAARRVSTSTIAYRPDSSFMKSIMA 323
gO-1b   267 KEQSEKKSKKSQSTTTPYSPYTTSTALNVTTNATYSVTTTARRVSTSTIAYRPDSSFMKSIMT 323
gO-1c   265 KEQLEKKTKKSQSTTTPYFSYTTSTALNVTTNATYKVTTSAKRIPTSTIAYRPDSSFMKSIMA 321
gO-2a   264 KEQLEKKTKKSQSTTTPYLSYTTSTALNVTTNVTYSVTTTAKRVPTSTIAYRPDSSFMKSIMA 320
gO-2b   266 KEQFEKKIKKSQSSTTPYFSYTTSTAPNVTTNVTYGVTTTARRVPTSTIAYRSDSSFMKSIMA 322
gO-3    268 KEP-QKKSKKSQSTTTPSF-YTTFTTFNV-TNVTYSATTTARRTPTSTIAYRPDSSFMKSIMT 321
gO-4    263 KSI-SKKSR--VSTTTPYSSYT-STIFNVSTNVTYSPIV-PTRIPTSTIGYRPDENFMKSILT 314
gO-5    273 KEQPQKKNKKSQSTTTPYLSYTTSTAFNVTTNVTYSATAAVTRVATSTTGYRPDSNFMKSIMA 329
```

(d)

```
gH-1      1 MRPGLPPYLTVFTVYLLSHLPSQRYGADAASEALDPHAFHLLLNTYGRPIRFLRENTTQCTYNSSLRNSTVVRENAISFNFFQSYNQYYV 90
gH-2      1 MRPGLPSYLIVLAVCLLSHLLSSRYGAEAISEPLD-KAFHLLLNTYGRPIRFLRENTTQCTYNSSLRNSTVVRENAISFNFFQSYNQYYV 89
```

Figure S1. Peptide sequence variations that define the genotypes of glycoproteins. Peptide sequence alignments were constructed using T-coffee version 13.45.0.4846264 [32]. Sequence alignments are color-coded: red indicates a highly conserved residue, blue a weakly conserved residue, and black a non-conserved residue; dashes indicate insertions/deletions. **(a)** The most variable regions defining the five gB genotypes are codons 26-70 and codons 441-490. The GenBank accession numbers of published reference genotype sequences are: gB-1 (Towne strain, ACM48044.1), gB-2 (AD169 strain, DAA00160.1), gB-3 (Toledo strain, ADD39116.1), gB-4 (C194 strain, AAA45925), and gB-5 (saliva isolate, AZB53144). **(b)** The most variable regions defining the four gN genotypes and their subtypes are codons 1-87. The GenBank accession numbers of published reference genotype sequences are: gN-1 (AD169 strain, P16795.1), gN-2 (Can 2 strain, AAL77763.1), gN-3a (PS strain, AAL77773.1), gN-3b (A8-27F strain, AAO24841.1), gN-4a (ZV strain, AAL77779.1), gN-4b (Towne strain, AGT36491.1), and gN-4c (Toledo strain, AAS48964.1). **(c)** The most variable regions defining the five gO genotypes and their subtypes are codons 1-98 and codons 270-313. The GenBank accession numbers of published reference genotype sequences: gO-1a (AD169 strain, ACL51143.1), gO-1b (Cincy 2strain, ACS93309.1), gO-1c (Toledo strain, AAS48965.1), gO-2a (FUK19U strain, ABY48952.1), gO-2b (SW1102 strain, AAN40063.1), gO-3 (SW5 strain, AAN40074.1), gO-4 (Towne strain, AGT36493.1), and gO-5 (Merlin strain, YP_081522.1). **(d)** The most variable regions defining the two gH genotypes are codons 1-37. The GenBank accession numbers of published reference genotype sequences are: gH-1 (AD169 strain, ACL51144.1) and gH-2 (Towne strain, AGT36494.1).
